# An analysis of correlations among four outcome scales employed in clinical trials of patients with major depressive disorder

**DOI:** 10.1186/1744-859X-8-4

**Published:** 2009-01-23

**Authors:** Qin Jiang, Saeeduddin Ahmed

**Affiliations:** 1 Wyeth Research, Collegeville, Pennsylvania, PA, USA

## Abstract

**Background:**

The 17-item Hamilton Depression Rating Scale (HAM-D_17_) remains the 'gold standard' for measuring treatment outcomes in clinical trials of depressed patients. The Montgomery Ǻsberg Depression Rating Scale (MADRS), Clinical Global Impressions-Severity (CGI-S) and -Improvement (CGI-I) scales are also widely used.

**Objective:**

This analysis of data from 22 double-blind, placebo-controlled clinical studies of venlafaxine in adult patients with major depressive disorder was aimed at assessing correlations among these 4 scales.

**Methods:**

Changes from baseline for MADRS, HAM-D_17 _and CGI-S, and end point CGI-I scores and response (≥50% decrease from baseline HAM-D_17 _or MADRS, or CGI-S or CGI-I score ≤2) were analysed. Pearson correlation coefficients were calculated for all pairs of the four scales (HAM-D_17_/MADRS, HAM-D_17_/CGI-S, HAM-D_17_/CGI-I, MADRS/CGI-S, MADRS/CGI-I, CGI-S/CGI-I) at different time points. Effect sizes were calculated using the Cohen *d*.

**Results:**

Correlations were significant at all time points (p < 0.0001), increased over the course of treatment, and were similar across treatment groups. Effect sizes ranged from 0.31 to 0.42; MADRS and CGI-I effect sizes were slightly greater compared with HAM-D_17 _or CGI-S for continuous measures and response.

**Conclusion:**

Although MADRS and CGI-I were more sensitive to treatment effects, HAM-D_17_, MADRS, CGI-S and CGI-I scores present a consistent picture of response to venlafaxine treatment.

## Background

Many instruments have been developed to measure outcomes in studies of patients with major depressive disorder (MDD). Among them, the Hamilton Depression Rating Scale (HAM-D) [1], the Montgomery Ǻsberg Depression Rating Scale (MADRS) [2], and the Clinical Global Impressions-Severity scale (CGI-S) and -Improvement scale (CGI-I) [3], are investigator-rated instruments; the CGI-I differs from the other three scales in that it assesses the degree of symptom improvement rather than absolute severity of symptoms or specific pathology [3]. The HAM-D and the MADRS scales measure depressive symptoms, whereas the CGI-S and CGI-I assess global outcome.

The HAM-D was developed in the 1950s to evaluate efficacy of first-generation antidepressants; the 17-item HAM-D (HAM-D_17_) has been accepted by many as the standard for measuring therapeutic efficacy in clinical trials [1]. However, one problem with the HAM-D is that individual items are often multidimensional, with poor inter-rater and retest reliability. As a result, the HAM-D total score can be ambiguous [4]. The MADRS was designed to address some of the limitations of the HAM-D. Specifically, the MADRS may be more sensitive to treatment-related changes in depression and may better distinguish responders from non-responders [2,5]. Recent analyses have confirmed the correlation between HAM-D, MADRS, and CGI-S in a systematic literature review and two retrospective chart reviews [4-6].

The present analysis was undertaken in a large dataset of 22 double-blind, placebo-controlled, clinical studies of venlafaxine in patients with MDD to identify and assess correlations among these 4 widely-used, rating scales: the HAM-D_17_, MADRS, CGI-S, and CGI-I.

## Methods

### Studies and patients

Data were pooled from 22 multicenter, double-blind, placebo-controlled studies of venlafaxine (Table 1). All studies included adult patients with MDD, defined according to the diagnostic criteria from the *Diagnostic and Statistical Manual of Mental Disorders *(DSM-III [7], DSM-III-R [8], or DSM-IV [9] depending on when the study was designed). Outpatients were enrolled in 19 studies [10-22] and inpatients were enrolled in the other 3 studies [23] [Wyeth Research: Data on File. Collegeville, PA, USA: Wyeth Research; 2006. unpublished data]. Two studies (016 and 206) enrolled patients with melancholia [10,23], and one study (360) enrolled patients with concomitant anxiety[21]. Study durations ranged from 4 weeks to 52 weeks.

**Table 1:** Summary of 22 placebo-controlled clinical trials of venlafaxine for treatment of major depressive disorder^a^

**Study no.**	**IR/ER**	**Fixed/flexible** **Dosing**	**Dose range (mg/day)**	**Practice setting**	**Duration (weeks)**	**Median baseline HAM-D**_17_
014 [11]	IR	Fixed	75, 150, 225	Outpatient	6	21
015 [12]	IR	Fixed	75, 150, 225	Outpatient	8	21
016 [10]	IR	Flexible	37.5 to 375	Inpatient	6	26
203 [16]	IR	Fixed ranges	75, 150 to 225, 300 to 375	Outpatient	6	22
206 [23]	IR	Flexible	150 to 375	Inpatient	4	27
208 [14]	IR and ER	Flexible	IR: 75 to 150; ER: 75 to 150	Outpatient	12	22
209 [15]	ER	Flexible	75 to 225	Outpatient	8	21
211 [13]	ER	Flexible	75 to 225	Outpatient	8	22.5
300	IR	Flexible	150 to 375	Inpatient	6	29
301	IR	Flexible	75 to 225	Outpatient	6	22
302 [17]	IR	Flexible	75 to 200	Outpatient	6	22
303 [18]	IR	Flexible	75 to 225	Outpatient	6	22
313 [19]	IR	Fixed ranges	25, 50 to 75, 150 to 200	Outpatient	6	23
341	IR	Flexible	100 to 200	Outpatient	52	22
342 [20]	IR	Fixed	75, 150, 200	Outpatient	12	22
343	IR	Fixed ranges	100 to 150, 175 to 225	Outpatient	14	20
360 [21]	ER	Flexible	75 to 225	Outpatient	12	25
367 [22]	ER	Fixed	75, 150	Outpatient	8	25
372	IR	Flexible	200 to 375	Outpatient	6	22
384	ER	Flexible	150 to 375	Outpatient	6	25
402	ER	Flexible	37.5 to 300	Outpatient	10	23
414	ER	Flexible	37.5 to 300	Outpatient	10	22

ER, extended release; HAM-D_17_, 17-item Hamilton Depression Rating Scale; IR, immediate release.

^a^Data on File at Wyeth Research. 2006.

Only data from patients receiving venlafaxine or placebo were included in this analysis, although 15 studies included an additional active-comparator arm [10-13,16-18,21] [unpublished data]. Venlafaxine extended release (ER) was used in 7 studies and venlafaxine immediate release (IR) in 14. In one trial, both formulations were used [14]. Venlafaxine IR was administered twice or three times daily in fixed or flexible doses ranging from 25 to 375 mg/d [11-14,16-20] [unpublished data]. Venlafaxine ER was administered once daily in fixed or flexible doses ranging from 37.5 to 375 mg/d [13-15,21,22] [unpublished data].

### Statistical analysis

Continuous outcomes were defined as total change from baseline for MADRS and HAM-D_17_, change in score from baseline for CGI-S, and end point scores for CGI-I. These scores were calculated using observed data for the total patient populations at weeks 1, 2, 3, 4, 6, and 8 (for studies less than 8 weeks in duration, data were included for the number of weeks available), and for the final on-therapy (FOT) visit. HAM-D_17_, MADRS, CGI-S, and CGI-I scores were stratified by treatment arm, and Pearson correlation coefficients were calculated for all possible pairs of the four scales (HAM-D_17 _vs MADRS, HAM-D vs CGI-S, HAM-D_17 _vs CGI-I, MADRS vs CGI-S, MADRS vs CGI-I, CGI-S vs CGI-I) for each of the data points.

The four scales also were used to determine binary outcomes (response or no response). For CGI-I and CGI-S, response was defined as scores ≤2, and for HAM-D_17 _and MADRS total scores, response was defined as a 50% or greater decrease from baseline. Pearson correlation coefficients were determined for all possible pairs of the four scales for binary outcomes at weeks 1 through 8. Correlations were calculated for the FOT scores for the total population, and separately for those in the venlafaxine and placebo arms.

Pearson product-moment correlation coefficient (*r*), a measure of the tendency of two variables to increase or decrease together, was used to measure the correlation of a pair of two efficacy variables measured on the same subject. Effect sizes (Cohen *d*) were calculated to measure the magnitude of the treatment effect at the FOT evaluation for the pooled data and individually for each study.

## Results

At baseline, 5,117 observations were available for the HAM-D_17_, 4,871 for the MADRS, and 5,103 for the CGI-S, respectively. Mean baseline scores were 23.0, 29.1, and 4.4 for HAM-D_17_, MADRS, and CGI-S, respectively. Pretreatment correlations were 0.52 (CGI-S and HAM-D_17_), 0.53 (CGI-S and MADRS), and 0.62 (HAM-D_17 _and MADRS).

**Figure 1:**
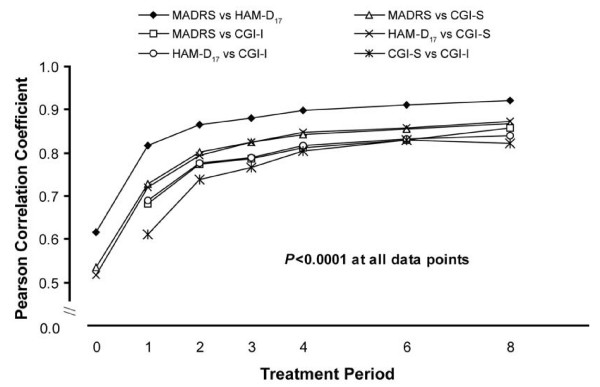
**Correlation coefficients, changes from baseline (all patients)**. CGI-I, Clinical Global Impressions Improvement scale; CGI-S, Clinical Global Impressions Severity scale; HAM-D17, 17-item Hamilton Rating Scale for Depression; MADRS, Montgomery Ǻsberg Depression Rating Scale.

**Figure 2:**
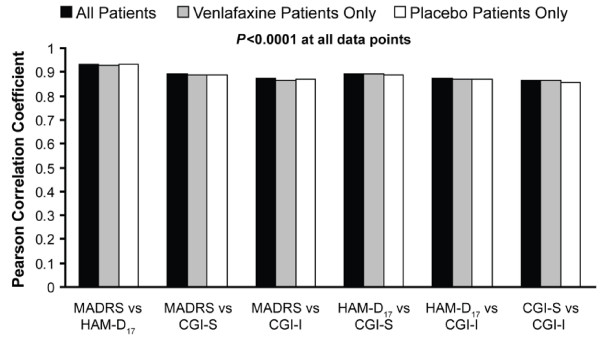
**Pearson correlation coefficient, changes from baseline (final on therapy)**. CGI-I, Clinical Global Impressions Improvement scale; CGI-S, Clinical Global Impressions Severity scale; HAM-D17, 17-item Hamilton Rating Scale for Depression; MADRS, Montgomery Ǻsberg Depression Rating Scale.

Correlations between scales were significant at all time points (p < 0.0001) and increased over the course of treatment. At week 8, correlations ranged from 0.82 (CGI-S and CGI-I) to 0.92 (HAM-D_17 _and MADRS) (Figure 1). Correlations for the FOT scores also were significant (p < 0.0001), ranging from 0.87 (CGI-S and CGI-I) to 0.93 (HAM-D_17 _and MADRS) (Figure 2). Comparisons were statistically similar for the total population, the venlafaxine group, and the placebo group.

**Figure 3:**
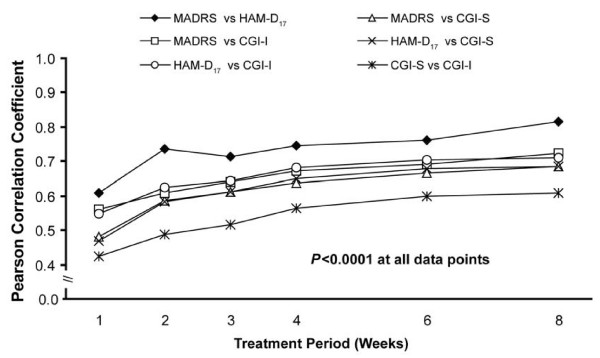
**Correlation between definitions of response (all patients)**. CGI-I, Clinical Global Impressions Improvement scale; CGI-S, Clinical Global Impressions Severity scale; HAM-D17, 17-item Hamilton Rating Scale for Depression; MADRS, Montgomery Ǻsberg Depression Rating Scale.

**Figure 4:**
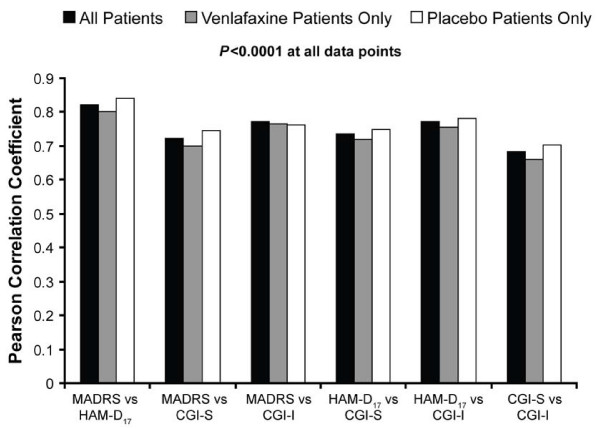
**Correlation between definitions of response (final on therapy)**. CGI-I, Clinical Global Impressions Improvement scale; CGI-S, Clinical Global Impressions Severity scale; HAM-D17, 17-item Hamilton Rating Scale for Depression; MADRS, Montgomery Ǻsberg Depression Rating Scale.

Correlation coefficients between binary outcomes (that is, response) were lower, ranging from 0.42 (CGI-I and CGI-S) to 0.61 (HAM-D_17 _and MADRS) at week 1 and from 0.61 (CGI-I and CGI-S) to 0.81 (HAM-D_17 _and MADRS) at week 8 (Figure 3). The correlations between binary outcomes at the FOT visit ranged from 0.68 (CGI-I and CGI-S) to 0.82 (MADRS and HAM-D_17_) (Figure 4). All correlation coefficients were significant at all data points (p < 0.0001).

**Figure 5:**
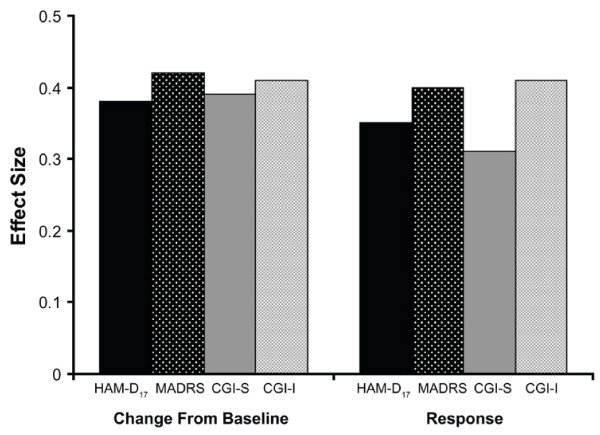
**Effect size for venlafaxine vs placebo (all patients, final on therapy)**. CGI-I, Clinical Global Impressions Improvement scale; CGI-S, Clinical Global Impressions Severity scale; HAM-D17, 17-item Hamilton Rating Scale for Depression; MADRS, Montgomery Ǻsberg Depression Rating Scale.

Pooled effect sizes for the continuous outcomes ranged from 0.39 on the CGI-I to 0.42 on the CGI-S (Figure 5). Effect sizes for the binary outcomes were lower, ranging from 0.31 (CGI-I response) to 0.41 (CGI-S response). Although differences were small, MADRS and CGI-I were better able to detect differences between venlafaxine and placebo than HAM-D_17 _or CGI-S for both sets of outcomes. Effect sizes across the individual studies varied considerably, but the pattern of results was largely consistent with that of the pooled data. In the majority of studies, effect sizes were greater on the CGI-I than CGI-S (continuous outcomes: 12 of 22 studies; response: 15 of 22 studies) and were greater on the MADRS compared with the HAM-D (continuous: 12 of 21 studies; response: 14 of 21 studies) (data not shown).

## Discussion

The data presented here, which are derived from a large pooled dataset from 22 clinical trials, confirm and expand results of earlier comparisons of these 4 commonly used depression rating scales [4-6]. Previous analyses have included data from samples that were smaller and rather homogeneous in terms of baseline depression severity and duration of treatment; these analyses evaluated treatment effects with a variety of antidepressants, including tricyclic antidepressants, selective serotonin reuptake inhibitors, and serotonin-norepinephrine reuptake inhibitors [5,6]. The trials in this analysis all included patients with MDD. However, the diagnostic criteria differed according to the DSM criteria accepted at the time individual studies were designed. All studies in this analysis used venlafaxine; however, they differed in the venlafaxine formulation used, dosing regimens (fixed or flexible), and duration of study treatment. The variability among the studies analysed here did not appear to confound the results, as the observations made using the HAM-D_17_, MADRS, CGI-S, and CGI-I were highly correlated. Furthermore, despite the differences between this and other analyses, the findings are consistent [6]. As might be expected, the highest correlations were between the HAM-D_17 _and the MADRS rating scales, which share several items, have similar modes of administration and rating, and are generally performed by the same clinician. However, in some clinical trials, depression rating assessments and assessments of global illness severity or improvement may be performed by different clinicians; this may have contributed to the lower correlations between the HAM-D_17 _or MADRS scales and the CGI scales observed in this analysis. The consistently and modestly lower correlations between the CGI-S and CGI-I scales were unexpected as these scales are sometimes considered interchangeable. However, this may be explained by the relatively narrow distribution of the score range (1 to 7) compared with the ranges for the HAM-D_17 _and MADRS total scores.

Although they were significant, correlation coefficients among binary outcomes based on the scales were lower than those for the change from baseline or FOT scores. Moreover, effect sizes were smaller for all scales in measuring the binary outcomes. These differences may be related to the definitions of response or no response that were used for the different scales. Some patients may have experienced significant improvement, which would be reflected in the change from baseline, although the scores did not meet the threshold for response.

## Conclusion

Overall, these results suggest that HAM-D_17_, MADRS, CGI-S, and CGI-I scores present a consistent picture of response to antidepressant therapy with venlafaxine.

## List of abbreviations

CGI-I/S: Clinical Global Impressions-Improvement/-Severity scale; DSM: Diagnostic and Statistical Manual of Mental Disorders; ER: extended release; FOT: final on-therapy; HAM-D_17_: 17-item Hamilton Depression Rating Scale; IR: immediate release; MADRS: Montgomery Ǻsberg Depression Rating Scale; MDD: major depressive disorder.

## Competing interests

QJ is an employee of Wyeth; SA is a former employee of Wyeth.

## Authors' contributions

Both authors contributed to the research and writing of this manuscript and were involved in the development of the statistical analysis plan. QJ performed the statistical analyses, both QJ and SA contributed to manuscript development and read and approved the final manuscript draft
